# Bioaccessible Phenolic Alkyl Esters of Wine Lees Decrease COX-2-Catalyzed Lipid Mediators of Oxidative Stress and Inflammation in a Time-Dependent Manner

**DOI:** 10.1021/acs.jafc.4c05086

**Published:** 2024-08-15

**Authors:** Concepción Medrano-Padial, Irene Pérez-Novas, Raúl Domínguez-Perles, Cristina García-Viguera, Sonia Medina

**Affiliations:** Laboratorio de Fitoquímica y Alimentos Saludables (LabFAS), CSIC, CEBAS, Campus Universitario de Espinardo 25, 30100 Espinardo, Murcia, Spain

**Keywords:** winery byproducts, lipophenols, simulated *in vitro* gastrointestinal digestion, digestive enzymes, prostanoids, anti-inflammatory activity

## Abstract

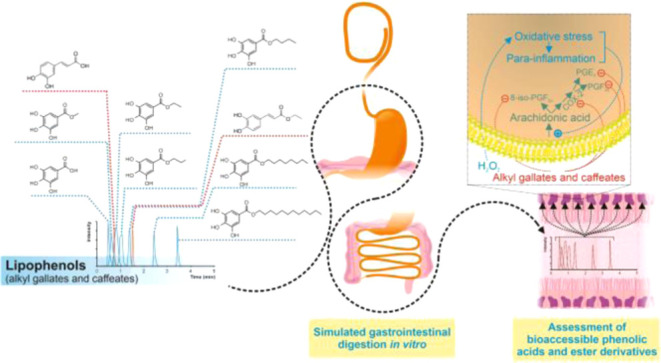

Lipophenols, phenolic compounds esterified with fatty alcohols or fatty acids, provide greater health benefits upon dietary ingestion of plant-based foods than unesterified (poly)phenols. Based on this premise, the present study aimed to demonstrate the role of gastrointestinal enzymes (pepsin, pancreatin, and pancreatic lipase) in releasing alkyl gallates and *trans*-caffeates from wine lees, providing bioactive compounds with enhanced capacities against oxidative stress (OS) and para-inflammation. The UHPLC–ESI-QqQ-MS/MS-based analysis revealed ethyl gallate and ethyl *trans*-caffeate as the most prominent compounds (1.675 and 0.872 μg/g dw, respectively), while the bioaccessibility of the derivatives of gallic and caffeic acids was dependent on the alkyl chain properties. The *de novo* formation of alkyl gallates during gastric and intestinal digestion resulted from intestinal enzyme activity. Moreover, the *in vitro* capacity of bioaccessible alkyl esters of gallic and *trans*-caffeic acids to reduce cyclooxygenase-2 concentration and modulate oxilipins related to OS (8-iso-PGF_2α_) and inflammation (PGF_2α_ and PGE_2_) was demonstrated in a time-dependent manner. In conclusion, the presence of alkyl esters of gallic and *trans*-caffeic acids in wine lees and their subsequent formation during digestion of this byproduct emphasize their value as a source of antioxidant and anti-inflammatory compounds, encouraging the consideration of wine lees as a valuable ingredient for health-promoting coproducts.

## Introduction

Lipophenols are phenolic compounds esterified with fatty alcohols or fatty acids, offering enhanced bioaccessibility and bioavailability compared to unesterified (poly)phenols; the extent of these enhancements varies based on the structural features of the alkyl chain.^1^ Eventual improvements of the biological properties are due to their amphiphilic characteristics that confer higher cell membrane affinity.^2^ This trait allows for augmented intracellular concentrations and, consequently, more powerful biological capacities,^2,3^ thus surpassing the constraints enclosed to the biological scope of (poly)phenols in terms of bioavailability.^3^ The bioactivities identified for lipophenols include anti-inflammatory, antihyperglycemic, antitumor, or antimicrobial capabilities.^4−6^ In addition, the capacity of lipophenols of gallic and *trans*-caffeic acids to prevent oxidative stress (OS) is especially relevant because of the mainstream nature of OS and its association with the course of several disabling diseases.^5,7,8^

In nature, lipophenols have been described in several plant materials. For instance, gallic acid and some galloyl esters are present in grape coproducts and byproducts.^2,9,10^ Wine lees are byproducts of special interest since they constitute a pollutant semisolid residue without an identified processing alternative toward added-value coproducts. However, this material constitutes a valuable source of bioactive phytochemicals, transformed by the metabolism of yeasts and lactic acid bacteria (LAB) during winemaking that renders new compounds,^11,12^ including lipophenols. To take advantage of the lipophenolic profile of winery byproducts, new valorization procedures addressed to convert wine lees into a sustainable source of healthy phytochemicals should include the biological contribution of these compounds.^13−15^ Beyond the biological power of phenolic acid alkyl esters, their actual biological interest *in vivo* for treating intestinal bowel disease (IBD) depends on their gastrointestinal extractability and stability under physicochemical conditions of the digestive system, which has been poorly evaluated. Thereby, additional studies are required to uncover the bioaccessibility of lipophenols and provide comprehensive information on changes in the chemical structure and bioactivity.

As previously reported for hydroxytyrosol (HT) esterified with fatty acids (HT-FAs), in addition to hydrolysis reactions during digestion,^16,17^ new esterifications could occur through these processes, modifying the initial quantitative lipophenolic profile.^17^ In this concern, the contribution of different digestion stages to the bioaccessibility of galloyl and *trans*-caffeoyl esters remains underexplored. To advance toward this objective, it is essential to unravel the effect of digestive factors on the biological scope of lipophenols in specific matrices since the evaluation of standards provides negligible results due to the food matrix effect.^18^

Based on these antecedents, the present work aims to determine the quantitative profile of gallic and *trans*-caffeic acids (3,4,5-trihydroxybenzoic acid and 3′,4′-dihydroxycinnamic acid, respectively), according to the standardized nomenclature for dietary (poly)phenol catabolites,^19^ and their alkyl derivatives (methyl, ethyl, propyl, butyl, octyl, and lauryl gallates, as well as ethyl *trans*-caffeate) in wine lees using advanced analytical techniques (UHPLC–ESI-QqQ-MS/MS). Moreover, the contribution of gastric and intestinal digestive phases, and the specific enzymes involved in each of them (pepsin, pancreatin, and pancreatic lipase), is studied regarding the contribution to the bioaccessibility of lipophenols. In addition, the ability of bioaccessible lipophenols to prevent OS and para-inflammation can be determined by evaluating the levels of cyclooxygenase-2 (COX-2) and isoprostanoid lipid mediators (prostaglandins (PGs) and isoprostanes (IsoPs)). To examine the contribution of individual lipophenols to the biological activities monitored, principal component analysis (PCA) was used.

## Materials and Methods

### Chemicals and Reagents

Standards of gallic and *trans*-caffeic acids and their alkyl derivatives (methyl, ethyl, propyl, butyl, octyl, and lauryl gallates, as well as ethyl *trans*-caffeate); the β-glucuronidase from *Helix pomatia* (type H-2); and digestive enzymes porcine pepsin (P6887), pancreatin from porcine pancreas (P7545, 8× USP), and lipase from porcine pancreas (L3126) were obtained from Sigma-Aldrich Co. (St. Louis, MO). Standards of prostanoids 8-iso-PGF_2α_, PGF_2α_, and PGE_2_ were purchased from Cayman Chemicals (Ann Arbor, MI). The COX-2 quantification kit was from Abcam (ab267646) (Cambridge, U.K.). Solid-phase extraction (SPE) cartridges (Strata X-AW) were provided by Phenomenex (Torrance, CA), and all LC–MS solvents were obtained from JT Baker (Phillipsburg, NJ). Ultrapure water was produced by using a Millipore water purification system (Bedford, MA). Trypsin-ethylenediaminetetraacetic acid (EDTA), Eagle’s minimum essential medium (EMEM), l-glutamine, fetal bovine serum (FBS), penicillin/streptomycin, and essential amino acids were from Gibco (ThermoFisher Scientific, Madrid, Spain), and the 24-well plates were from Corning (New York).

#### Plant Material

Wine lees of *Vitis vinifera* L. var. “Monatrel” were collected in 2022 after the vinification process from Bodegas ViñaElena S.L. (Jumilla, Murcia, Spain). For analytical purposes, this byproduct was dried up in an oven to a constant weight by applying a descendent temperature gradient (from an initial temperature of 75 °C to a final temperature of 60 °C over 10 h). The dry material was then ground to a fine powder, stored, and protected from light for further phytochemical extraction and *in vitro* simulated gastrointestinal digestion.

### *In Vitro* Simulated Gastrointestinal Digestion

Gastrointestinal digestions were performed on dehydrated wine lees powder following the simulated static *in vitro* digestion method described in the literature,^20,21^ with minor modifications.^22^ The contribution of separate digestion stages to the quantitative profile of the bioaccessible fraction of wine lees was addressed by performing isolated gastric and intestinal digestions. Moreover, complete gastrointestinal digestion was done. The digestions were carried out in the presence/absence of pepsin, pancreatic, and pancreatic lipase to determine their relative contribution to the bioaccessibility of phenolic acids and lipophenols. After digestion, the digested samples were centrifuged at 1600*g* for 5 min at 4 °C to separate the bioaccessible fraction from the residual material. The former was filtered through a 0.22 μm poly(vinylidene fluoride) (PVDF) filter (Millipore, MA) and frozen at −80 °C until UHPLC–QqQ-MS/MS analysis.

### Cell Line, Culture, and Experiment Conditions

The human colon adenocarcinoma (Caco-2, TCCHTB37) cell line was obtained from the American Type Culture Collection (ATCC, Rockville, MD) (passage number between 15 and 20). Caco-2 cells were grown following the methodology described to obtain a monolayer model of the intestinal barrier.^23^ Cells were allowed to adhere for 24 h. Afterward, the culture medium was replaced by an FBS-free medium supplemented with 10% of the bioaccessible fraction of wine lees. The control cells were exposed to culture media supplemented with an equal volume of the diluted blank of digestion. After 24 h of exposure, OS was induced by treating cells with 50 μM H_2_O_2_ (final concentration). Both the supernatant and detached cells were collected after 1, 6, 12, and 24 h of exposure to the pro-oxidant environment (in the presence/absence of bioaccessible lipophenols) and centrifuged at 5000 rpm for 10 min. Cell viability was determined by the trypan blue exclusion assay to monitor the cytotoxic effects of the bioaccessible fraction, upon evaluating different supplementation ratios (1:3, 1:5, and 1:10, v/v) of the culture media. The two former ratios induced a lack of viability and adhesion ability of cells. Thereby, the supplementation ratio used to evaluate the capability of bioaccessible lipophenols to modulate the concentration of COX-2 and derived oxilipins was 1:10 (v/v). The pellets and supernatants were separated and kept at −80 °C for the assessment of COX-2 (culture supernatants) and the content of OS and inflammation-related oxylipins (8-iso-PGF_2α_, PGF_2α_, and PGE_2_) (growth media) by the enzyme-linked immunosorbent assay (ELISA) and UHPLC–ESI-QqQ-MS/MS analyses, respectively.

### Assessment of Alkyl Esters of Gallic and *trans*-Caffeic Acids by UHPLC–ESI-QqQ-MS/MS

**Figure 1:**
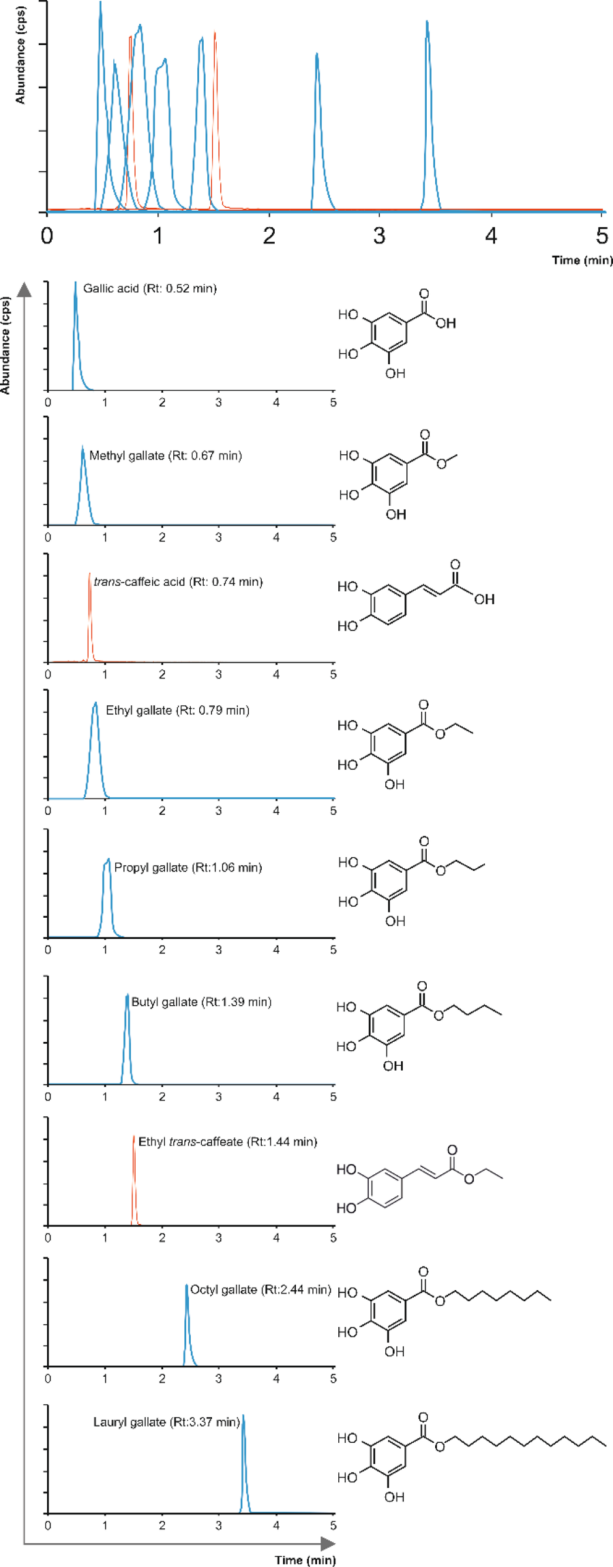
Representative UHPLC–ESI-QqQ-MS/MS chromatograms of phenolic acid and ester derivatives; cps, charges per second.

The chromatographic separation of gallic and *trans*-caffeic acids and their alkyl esters was performed using a UHPLC coupled with a 6460-triple quadrupole-MS/MS system (Agilent Technologies, Waldbronn, Germany), employing the chromatographic column BEH C18 1.7 μm (2.1 × 50 mm^2^) (Waters, Milford, MA), maintained at 30 °C, according to the methodology previously reported.^2^ The concentrations of galloyl and caffeoyl esters were calculated according to standard curves, which were freshly prepared with authentic standards each day of analysis (Figure 1 and Table S1). For data acquisition and processing, MassHunter software version B.08.00 (Agilent Technologies, Waldbronn, Germany) was used.

### Assessment of the COX-2 Activity in Epithelial Cells of the Intestine

The supernatants obtained from the Caco-2 culture following the treatments specified above (Cell Line, Culture, and Experimental Conditions section) were assessed for COX-2 activity using specific ELISA kits according to the manufacturer’s instructions (Abcam, Cambridge, U.K.).

### Extraction and UHPLC–ESI-QqQ-MS/MS Analysis of Cell Prostanoids

To determine the selected prostanoids, supernatants were processed according to the methodology described in the literature.^24^ The cleanup of the growth media by SPE was achieved using Strata X-AW cartridges, according to the procedure described by Medina et al.^25^ The eluted compounds were dried resorting to the SpeedVac concentration (Savant SPD121P, Thermo Scientific, MA), then reconstituted with deionized Milli-Q/0.1% formic acid in ACN (90:10, v/v), and filtered through a 0.22 μm PVDF filter (Millipore, MA). Subsequently, the concentrations of 8-iso-PGF_2α_, PGF_2α_, and PGE_2_ were analyzed using UHPLC–ESI-QqQ-MS/MS (Agilent Technologies, Waldbronn, Germany) by applying the methodology and settings described in the literature^25,26^ through the analysis of the parent masses, specific fragmentation patterns, and the retention time compared to authentic standards.^25,26^ The concentrations of prostanoids were calculated according to standard curves freshly prepared each day of analysis. Data acquisition and processing were performed using MassHunter software version B.08.00 (Agilent Technologies, Waldbronn, Germany).

### Statistical Analyses

All experimental conditions were performed in triplicate (*n* = 3), and the data were expressed as the mean ± standard deviation (SD). According to the normal distribution and homogeneity of variance of the data (determined by Shapiro–Wilk (<50 samples) and Levene tests, correspondingly), the obtained results were subjected to one-way analyses of variance (ANOVA); when statistical differences were identified, the variables were compared using Tukey’s multiple range test. Significant differences were set at *p* < 0.05.

Relationships between the concentration of bioactive phytochemicals quantified in the separate samples and their ability to protect against OS and para-inflammation by modulation of oxylipin levels were analyzed by PCA, a pattern recognition unsupervised classification method. Significant correlations were set at *p* < 0.05.

All statistical analyses were conducted using the SPSS 27.0 software package (LEAD Technologies, Inc., Charlotte, NC).

## Results and Discussion

### Bioaccessibility of Alkyl Esters of Gallic and *trans*-Caffeic Acids in Wine Lees

Since the biological activity of phytochemicals in plant-based foods depends on the concentration achieved in target cells, the ability of gastrointestinal digestion to extract galloyl and *trans*-caffeoyl alkyl esters into the intestinal lumen in close contact with epithelial cells and their stability under the digestive physicochemical and enzymatic conditions deserve to be unraveled.

#### Quantitative Profile of Alkyl Esters of Gallic and *trans*-Caffeic Acids in Wine Lees

**Figure 2:**
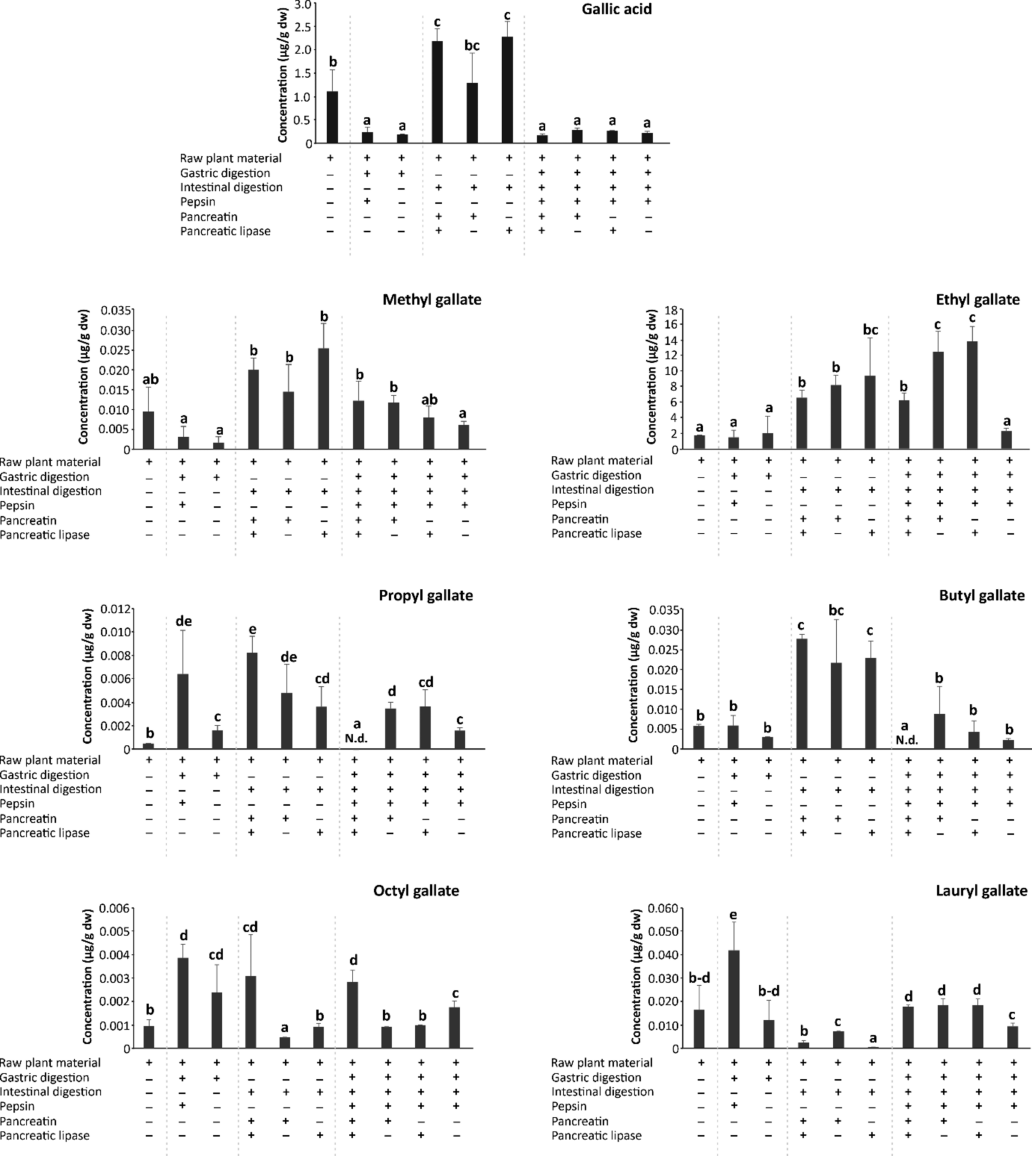
Bar plots showing the evolution of the concentrations (μg/g dry weight (dw)) of gallic acid and galloyl esters in wine lees and the gastric, intestinal, and gastrointestinal simulated *in vitro* digestion products with the specific application of the digestive enzymes, pepsin, pancreatin, and/or pancreatic lipase. Distinct lowercase letters within each bar plot indicate values significantly different at *p* < 0.01 according to one-way analyses of variance (ANOVA) and the multiple range test of Tukey (*n* = 3).

**Figure 3:**
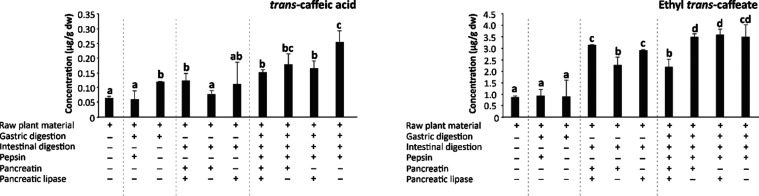
Bar plots showing the evolution of the concentration (μg/g dry weight (dw)) of *trans*-caffeic acid and ethyl *trans*-caffeate in wine lees during gastric, intestinal, and gastrointestinal simulated *in vitro* digestion with the specific application of digestive enzymes pepsin, pancreatin, and/or pancreatic lipase. Distinct lowercase letters indicate values significantly different at *p* < 0.01 according to one-way analyses of variance (ANOVA) and the multiple range test of Tukey (*n* = 3).

To establish the bioaccessibility of alkyl gallates and alkyl *trans*-caffeates in wine lees, it is essential to profile their concentration in the undigested material as a reference frame.^2^ Thus, ethyl gallate was the most abundant compound in wine lees, with a concentration of 1.675 μg/g dry weight (dw), while the concentration of unesterified gallic acid was 1.099 μg/g dw. The concentrations of other alkyl derivatives of gallic acid decreased in the following order: lauryl gallate (0.016 μg/g dw) > methyl gallate (0.010 μg/g dw) > butyl gallate (0.006 μg/g dw) > octyl gallate (0.001 μg/g dw) > propyl gallate (0.001 μg/g dw) (Figure 2). Furthermore, the concentration of *trans*-caffeic acid was 0.063 μg/g dw. In comparison, its ethyl derivative was found at a much higher level (0.872 μg/g dw) (Figure 3).

Comparing these results with previous descriptions in the literature, the concentration of phenolic acids was reported to be lower than that previously described for wine lees.^27^ These differences can be associated with preharvest factors, such as the variety and sensitivity to agroclimatic conditions, ripening stage, or characteristics of the production process, among others, which impact the secondary metabolism of plants.^27^ Beyond this, Souza da Costa et al. described that different grape tissues provide variable concentrations of alkyl esters of phenolic acids, whose presence in grape seeds, for instance, ranged between 0.86 and 34.47 mg/kg.^9^ Nonetheless, these differences seem to be due, beyond preharvest factors or plant physiology issues, to processing conditions. Hence, it should be noted that wine lees are residues formed from the original foodstuff (must and grape pomace) during the winemaking (fermentation) process, resulting from the metabolisms of yeast and LAB, which would alter the original lipophenolic content.^2,28^ In this concern, previous reports have described a concentration of gallic acid lipophenols (e.g., ethyl gallate) in the range 1.2–2.5 μg/g,^11^ which is in line with the results obtained in the present study, thus supporting the interest of wine lees as a source of ester derivatives of phenolic acids.

#### Effect of Gastrointestinal Digestion on the Quantitative Profile of Alkyl Esters of Gallic and *trans*-Caffeic Acids in Wine Lees

To establish the value of wine lees as a dietary source of galloyl and *trans*-caffeoyl alkyl esters and its potential application in the field of nutraceutical or functional ingredients, the extraction efficiency of gastrointestinal digestion and the stability of the released lipophenols should be analyzed.^29^ Gathering comprehensive information on these issues, as well as concerning eventual *de novo* synthesis of lipophenols of gallic and *trans*-caffeic acids present in wine lees, as already described on HT-FAs,^16^ would provide thoughtful insights into the health benefits of these bioactive phytochemicals.

The bioaccessibility after *in vitro* gastrointestinal digestion (including the complete set of digestive enzymes, namely, pepsin, pancreatin, and pancreatic lipase) of gallic and *trans*-caffeic acids ranged between 0.159 and 0.152 μg/g dw. However, only methyl, ethyl, octyl, and lauryl gallates and ethyl *trans*-caffeate were detected at concentrations higher than the limit of quantification (0.012, 6.207, 0.003, 0.018, and 2.197 μg/g dw, respectively) (Figures 2 and 3), suggesting variable release and stability of separate alkyl ester derivatives into the intestinal lumen. In this concern, the digestive extractability of the target phytochemicals has been associated with various factors, such as the presence of additional macromolecules in the food matrix and the reactivity of the diverse compounds according to the specific properties of the alkyl chain.^16,30^ In this regard, for instance, previous analyses of HT bioaccessibility have demonstrated a negative correlation between the unsaturation degree of the alkyl moiety and the efficiency of the hydrolysis reactions occurring during digestion.^31^ However, to transfer the gathered knowledge to a physiological dimension, these digestive reactions must be performed using authentic standard solutions and plant materials that provide complex mixtures of molecules.^16^ In addition, other factors, such as the interconversion of lipophenols one to another or *de novo* synthesis of alkyl esters from gallic and *trans*-caffeic acids upon their reaction with alcohols present in the matrix, can affect their final bioaccessibility. These reactions have already been demonstrated using an olive oil solid byproduct (alperujo), which indicates the interference of sugars and fibers with lipophenol bioaccessibility.^32^

In comparison with previous data on this concern, the reduced extraction of propyl and butyl gallates is consistent with diverse hypotheses: first, the influence of specific physicochemical conditions and pepsin activity to extract lipophenols in wine lees and, second, the lability of the monitored analytes against these conditions, according to the chemical properties of their alkyl chains.^33^ Thus, our findings fit well with previous studies reporting that concentrations of butyl and myristyl gallates gradually decrease in the gastric lumen.^34^

On the contrary, the rising concentrations recorded for ethyl and octyl gallates and ethyl *trans*-caffeate are in agreement with a recent study on *in vitro* gastrointestinal digestion of seeds of cupuassu (*Theobroma gradiflorum*), which described a significantly higher concentration of ethyl gallate after digestion (41.181 μg/g dw) compared to undigested samples, in which ethyl gallate was not detected.^35^ Similarly, this fact was reported in previous studies on the bioaccessibility of lipophenols (e.g., esters of HT).^16,36^ This may be due to the interaction of gallic and *trans*-caffeic acids with alcohols present in wine lees, forming ester derivatives through enzymatic or chemical mechanisms. The occurrence of these esterification reactions is supported by the evidence showing a reduced bioaccessibility of gallic and *trans*-caffeic acids,^37^ which are substrates of the esterification reactions. Moreover, these reactions mirror the modification of the phytochemical profile relative to that already described in wine lees and, thereby, its biological scope. This depends on the bioactivity of bioaccessible phenolic acids and alkyl esters and the synergies between individual compounds.

These results provide complementary evidence to previous descriptions of the bioaccessibility of lipophenols, resorting to the digestion of isolated compounds or authentic standards. In this concern, beyond assessing the resistance or sensitivity of the compounds against physicochemical and enzymatic conditions of gastrointestinal digestion (mainly hydrolysis reactions),^38−40^ the present work contributes to understanding the actual bioaccessibility of galloyl and *trans*-caffeoyl esters from complex matrices.

#### Contribution of the Gastric and Intestinal Digestion Phases and Related Enzymes to the Bioaccessibility of Alkyl Esters of Gallic and *trans*-Caffeic Acids in Wine Lees

To gain further insight into the contribution of separate digestion stages to the bioaccessibility of alkyl esters of gallic and *trans*-caffeic acids, gastric and intestinal digestions were assessed separately.^18,19^ Furthermore, these simulations were developed in the presence/absence of pepsin, pancreatin, and pancreatic lipase to assess the involvement of different digestive enzymes in the extractability (and eventual synthesis) of gallic and *trans*-caffeic acid esters (Figures 2 and 3).

The analysis of the chemolike fluid resulting from gastric digestion revealed different behaviors for gallic and *trans*-caffeic acids in this stage. Thus, while the gastric phase extracted ca. 20.9% gallic acid from wine lees, the *trans*-caffeic acid bioaccessibility was 93.1% (Figures 2 and 3). Similar performance to gallic acid was observed for methyl and ethyl gallates, with extraction efficiencies during gastric digestion ranging between 33.3% and 85.7% (Figure 2). In contrast, this digestion stage led to the efficient release of butyl gallate and ethyl *trans*-caffeate, resulting in the release of almost all their amounts from wine lees (104.5%, on average). Finally, propyl, octyl, and lauryl gallates achieved bioaccessibilities that were 2.6–12.5-fold higher than those in wine lees (Figure 2). These increases indicated that the release of gallic and *trans*-caffeic acid alkyl esters during gastric digestion is partially independent of the length of the alkyl chain of alcohol.

According to previous descriptions in the literature, the low release of methyl and ethyl gallates during gastric digestion could be associated with the physicochemical conditions in the gastric lumen, reactive macromolecules, and pepsin activity.^13,29,30^ However, the specific chemical properties of the different lipophenols seem to underlie their bioaccessibility, as some gallic and *trans*-caffeic acid lipophenols exhibited increased gastric concentrations (Figure 2). These increases might be associated with the proteolytic activity of pepsin, which raises concentrations of free arginine in the gastric compartment and, as a result, enhances the aqueous solubility of alkyl gallates through its interaction with their aromatic moiety.^41^ This hypothesis is supported by the decrease of lipophenol bioaccessibility in the absence of pepsin, by 25.8%, on average (Figures 2 and 3). The variations observed support the intricate interplay among food composition, enzymatic activity, and the phytochemicals’ structure and availability in the gastric environment.

When assessing the contribution of intestinal digestion to the overall bioaccessibility of alkyl gallates and *trans*-caffeates, a 1.9–15.9-fold increase in the concentrations of gallic and *trans*-caffeic acids, as well as methyl, ethyl, propyl, butyl, and octyl gallates and ethyl *trans*-caffeates, was observed compared to undigested wine lees (Figures 2 and 3). This increase was especially noticeable for propyl gallate, suggesting behaviors that might be associated with esterification of these phenolic acids with alcohols present in wine lees. However, the alcohol content (e.g., ethanol) of wine lees varies depending on a variety of factors, such as the sugar content of grapes, the type of wine, or the characteristics of the fermentation process,^42,43^ which can influence the formation of new ester derivatives. Nonetheless, the development of these reactions is linked to the putative role of pancreatic lipase, which catalyzes both hydrolysis and esterification reactions.^44^ The decrease in the amount of lauryl gallate in the simulated intestinal phase was an exception to the general trend and suggests that the esterification efficiency is associated with the carbon chain length, a factor that also influences the efficiency of hydrolysis reactions in the intestine.^1^

Simulated intestinal digestions were conducted in the presence and absence of both enzymes to understand the relative contribution of pancreatin and pancreatic lipase to the increased bioaccessibility of galloyl and *trans*-caffeoyl esters (Figures 2 and 3). As a result, the enzymatic activity of pancreatic lipase, which catalyzes hydrolysis, esterification, *trans*-esterification, and alcoholysis reactions,^45^ was found to be essential for obtaining high concentrations of gallic and *trans*-caffeic acid alkyl esters; when acting alone, pancreatic lipase produced higher concentrations of ethyl gallate and *trans*-caffeate (5.6 and 3.4 times higher than undigested wine lees, respectively), and in combination with pancreatin, it increased the levels of ethyl, propyl, butyl, and octyl gallates and ethyl *trans*-caffeate (3.9, 15.9, 4.8, 3.2, and 3.6 times higher content than undigested wine lees, respectively).

In terms of pancreatin, its enzymatic activity was critical for obtaining highly bioaccessible ethyl, propyl, and butyl gallates, as well as ethyl *trans*-caffeate (with concentrations 4.9, 9.4, and 3.7, and 2.6-fold higher than those in undigested wine lees, respectively) (Figures 2 and 3). Interestingly, these efficiencies are reflected in the significant differences recorded between the physiological combination of enzymes and the application of individual pancreatin and pancreatic lipase. Thus, the combined enzyme conditions provided higher bioaccessibility for ethyl, propyl, butyl, and octyl gallates and ethyl *trans-*caffeate. Nonetheless, digestions with only one enzyme yielded higher amounts of ethyl and propyl gallates, as well as ethyl *trans*-caffeates for both pancreatin and pancreatic lipase considered individually, and butyl gallate concerning pancreatic lipase (Figures 2 and 3). These efficiencies have been associated with the chemical properties of the alkyl chain, particularly the number of carbons. Indeed, this trait affects the special configuration and accessibility of the hydrolytic lipase enzymes.^46^ However, for gallic and *trans*-caffeic alkyl esters, the highest relevance of pancreatic lipase previously described for the formation of HT-FAs^16^ was not so pronounced, highlighting the relevance of the structural properties of the alkyl chain (e.g., the presence of double bonds or chain length) for enzymatic selectivity and efficient activity.

In addition to chemical/enzymatic digestion, the role of intestinal microbiota metabolism in the bioavailability and absorption of lipophenols not absorbed in the small intestine should not be overlooked. Indeed, microbiota metabolism could be key for the metabolism of lipophenols.^34^ However, the microbial involvement in the metabolization of these amphiphilic molecules is still underexplored. In this concern, to date, it has been described that, *in vivo*, alkyl derivatives of gallic acid can reach the cecum and colon, where they exert beneficial activities on intestinal health.^34^

The bioaccessibility of lipophenols of gallic and *trans*-caffeic acids from complex matrices like wine lees is critical to identifying the most active compounds against intestinal inflammation since the quantitative profiles obtained in the intestinal lumen can prevent OS and para-inflammation.

### Capability of Bioaccessible Phenolic Alkyl Esters to Modulate COX-2 Expression, Oxidative Stress, and Para-Inflammation-Related Prostanoids

Unraveling the capability of gallic and *trans*-caffeic acid alkyl esters to tackle the transversal molecular mechanisms involved in various degenerative pathologies and aging requires the assessment of various markers and mediators such as COX-2 and related isoprostanoids associated with OS (8-iso-PGF_2α_) and inflammation (PGF_2α_ and PGE_2_).^47^

Cycloxygenase (COX) is an enzyme that regulates the inflammatory process and synthesizes bioactive lipids by catalyzing the first steps in the synthesis of PGs and IsoPs.^48,49^ This enzyme presents two isoforms (COX-1 and COX-2) responsible for sequential reactions toward PGH_2_. This prostaglandin is metabolized by downstream enzymes, yielding the family of PG involved in the inflammatory process (PGF_2α_ and PGE_2_).^50^ Given the involvement of both COX isoforms in the inflammation process (pain, fever, and tumorogenesis), COX-1 and COX-2 have been recognized as molecular targets for the identification of new anti-inflammatory compounds.^49^ However, COX-2 is responsible for the production of prostanoids more closely related to the pathophysiology of inflammation (by triggering molecular mechanisms involving inflammatory signals, interleukins, and growth factors^51^) and is therefore the best choice to assess the anti-inflammatory potential of bioaccessible galloyl and *trans*-caffeoyl esters.

**Figure 4:**
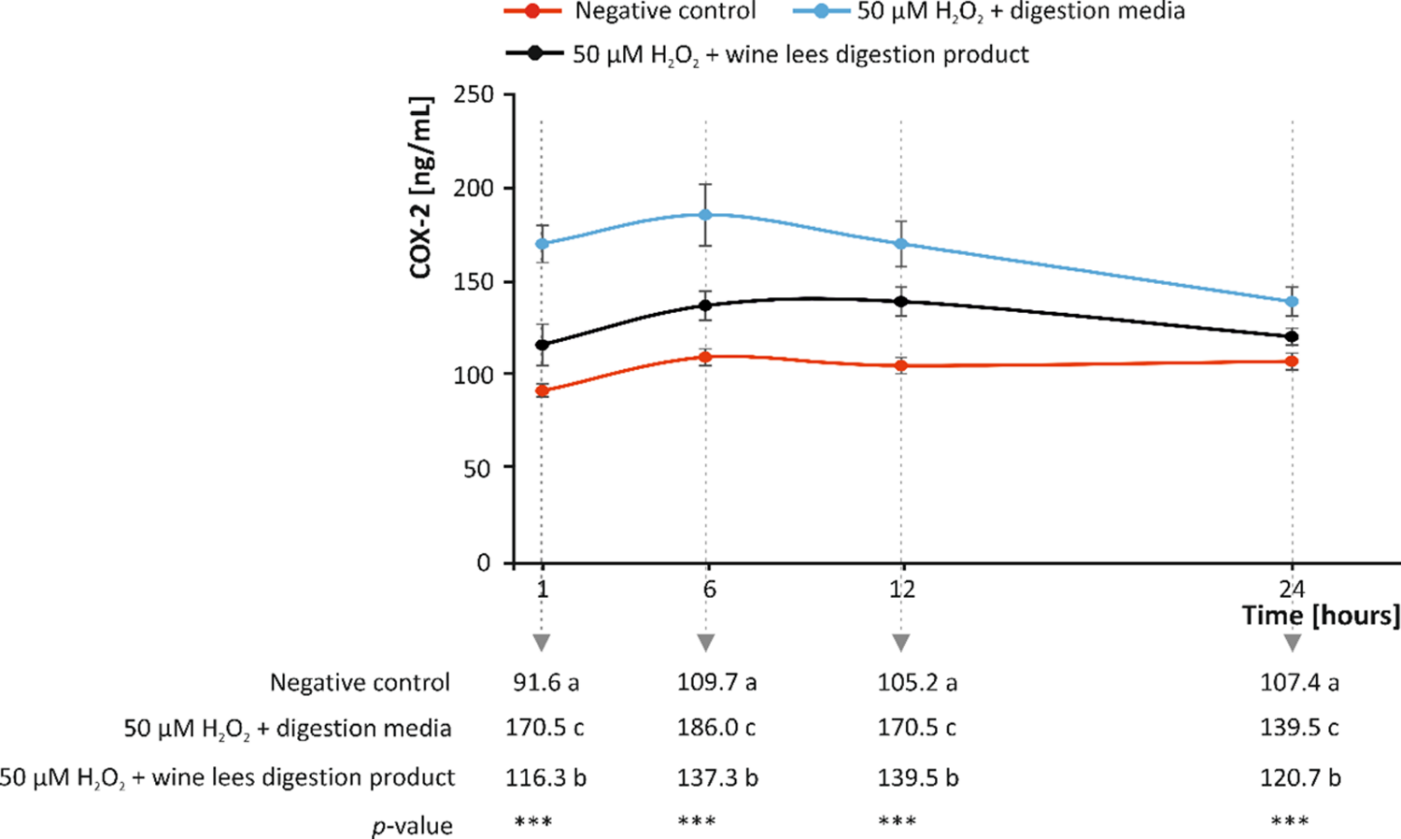
Modulation of the cyclooxygenase-2 (COX-2) concentration (ng/mL) by bioaccessible phenolic acids and phenolic esters of wine lees recorded at 1, 6, 12, and 24 h after the oxidative stimulus in the absence and presence of plant-material digestion products. Distinct lowercase letters indicate values significantly different at *p* < 0.001(***) according to one-way analyses of variance (ANOVA) and the multiple range test of Tukey (*n* = 3).

According to these premises, for the evaluation of the ability of bioaccessible alkyl esters of gallic and *trans*-caffeic acid to prevent inflammation associated with OS in the intestinal epithelium, after the development of a monolayer model intestinal barrier *in vitro*, the cells were exposed to a pro-oxidant environment characterized by active signaling pathways involved in inflammatory processes, including the upregulation of COX-2.^52^ Thereby, in the present study, 50 μM H_2_O_2_ was applied to the culture media to induce a para-inflammation phenotype secondary to OS in Caco-2 cells (positive control) and the subsequent increase in the COX-2 level (up to 166.6 ng/mL, on average) at sampling times 1, 6, 12, and 24 h after H_2_O_2_ application. This concentration of COX-2 significantly surpassed the level recorded in basal cells grown without a pro-oxidant stimulus (103.5 ng/mL, on average) (Figure 4). The response to pro-oxidant conditions allowed identifying the highest amount of COX-2 at 6 h after the application of the pro-oxidant stimulus, followed by a time-dependent decrease, which is in good agreement with previous descriptions in the literature.^53^ Alternatively, when epithelial cells were pretreated with the bioaccessible fraction of wine lees, which is rich in galloyl and *trans*-caffeoyl alkyl esters, the increase in COX-2 concentration was prevented at all monitored time points, resulting in values (116.3–139.5 ng/mL) significantly lower (*p* < 0.001) than the positive control (Figure 4).

This scenario fits well with the results obtained in research that has explored the underlying mechanisms through which bioactive phytochemicals (especially concerning lipophenols) develop the inhibition described here (Figure 4). Specifically, the capability of methyl gallate, among other synthetic alkyl esters of phenolic acids, to downregulate the expression of genes encoding for COX-2 has been demonstrated. Moreover, previous mechanistic studies have pointed out the ability of galloyl alkyl esters to hinder the signaling pathway of NF-κB, which is the main mechanism responsible for the COX-2-based anti-inflammatory effects.^50^ Beyond its effect on NF-κB, this molecular pathway is also associated with p38 mitogen-activated protein kinase (MAPK). Interestingly, both mechanisms contribute to fine-tuning other proinflammatory interleukins, such as TNF-α, IL-1β, and IL-6.^54,55^ This demonstrates that inflammation involves specialized substances (e.g., eicosanoids), which interfere with the secretion of proinflammatory interleukins.^56^

Since COX-2 is involved in the production of oxylipins through the oxygenation of arachidonic acid^57^ and because of their role as key mediators of OS and para-inflammation, which are linked with the production of the interleukins mentioned previously, the modulation of isoprostanoids, such as 8-iso-PGF_2α_ (OS-related isoprostanoid), and prostanoids like PGF_2α_ and PGE_2_ (inflammation-related) by the gallic and *trans*-caffeic acid alkyl esters from wine lees was analyzed.

**Figure 5:**
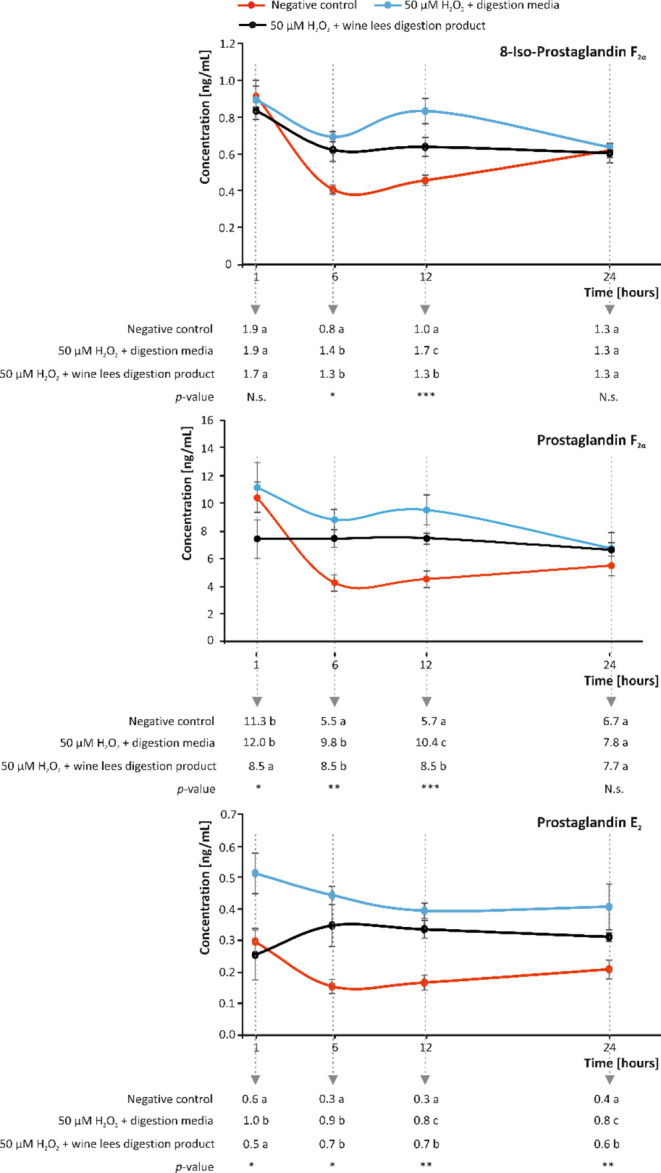
Modulation of the concentration (ng/mL) of isoprostane 8-Iso-PGF_2α_ and prostaglandins PGF_2α_ and PGE_2_, recorded at 1, 6, 12, and 24 h after oxidative stimulus in the absence and presence of plant-material digestion products. Distinct lowercase letters indicate values significantly different at *p* < 0.05 (*), *p* < 0.01 (**), and *p* < 0.001 (***), according to one-way analyses of variance (ANOVA) and the multiple range test of Tukey (*n* = 3). N.s., not significant. Modulation of the concentration (ng/mL) of isoprostane 8-Iso-PGF_2α_ and prostaglandins PGF_2α_ and PGE_2_, recorded at 1, 6, 12, and 24 h after oxidative stimulus in the absence and presence of plant-material digestion products. Distinct lowercase letters indicate values significantly different at *p* < 0.05 (*), *p* < 0.01 (**), and *p* < 0.001 (***), according to one-way analyses of variance (ANOVA) and the multiple range test of Tukey (*n* = 3). N.s., not significant.

In this regard, the environment generated when cells were exposed to 50 μM H_2_O_2_ provided twofold higher concentrations of 8-iso-PGF_2α_ than the negative control at 6 and 12 h after applying the pro-oxidant stimulus (Figure 5). Nonetheless, in cells pretreated with bioaccessible galloyl and *trans*-caffeoyl alkyl esters, this increase was significantly prevented, decreasing the 8-iso-PGF_2α_ concentration between 10% and 25% in a time-dependent manner (Figure 5). In addition to OS, the addition of H_2_O_2_ led to an increase in the PGs involved in the parainflammatory syndrome (PGF_2α_ and PGE_2_). Thus, while the PGF_2α_ concentration increased by 5.9–45.0%, depending on the sampling time considered, as a result of the pro-oxidant stimulus, the increase in PGE_2_ concentration was also significant, reaching up to 65% higher after the H_2_O_2_ treatment (Figure 5). When pretreating cells with bioaccessible alkyl gallates and caffeates from wine lees, it was found that they prevented the increase in both PGF_2α_ and PGE_2_ inflammatory markers and mediators by 15.0 and 28.0%, on average, respectively. This modulatory effect highlighted the valuable contribution of bioaccessible phenolic acid alkyl esters from wine lees to resolving inflammation mediated by inhibiting COX-2. In this sense, a recent study reported that ethyl gallate markedly increased the plasma level of PGE_2_ both *in vitro* and *in vivo* in a time-dependent manner, exerting this effect through the activation of the peroxidase active site of COX-1 and COX-2.^58^ However, it is important to consider that PGE_2_, through binding to different E-type prostanoid receptors EP1–EP4, may regulate the function of many cell types, leading to pro- and anti-inflammatory effects.^59^

In this concern, the ability of bioactive lipophenols to modulate the secretion of PGs via COX-2 modulation can influence the course of para-inflammation, according to the already described relationship between PGs and the expression of inflammatory interleukins, for instance, through IL-1β-induced cell dysfunction. However, the molecular mechanism underlying such association remains unclear.^60^ These molecular interactions have been described in the inflammatory processes associated with type 2 diabetes mellitus (T2DM), demonstrating the utility of tackling the IL-1β/COX-2/PGE_2_ pathway loop for preventing inflammation secondary to other metabolic processes, such as T2DM or OS.^61,62^ To some extent, the modification of the inflammation course by PGF2α is carried out through its potential to regulate the interleukin profile.^63^ Indeed, it has been demonstrated that this PG suppresses IL-1β, IL-6, and TNF-α, thus contributing to tackling the inflammatory cascade.^64,65^ This modulation capacity would reinforce the one already described for the IL-1β/COX-2/PGE_2_ pathway loop, further demonstrating the anti-inflammatory potential of bioaccessible galloyl and *trans*-caffeoyl alkyl esters. Indeed, bioaccessible molecules that interrupt the molecular pathways associated with PGF_2α_ and PGE_2_ offer a potential therapeutic strategy to enhance cell function in intestinal bowel disease, thus highlighting the potential of dietary interventions in preventing OS and inflammation.

### Principal Component Analysis

**Figure 6:**
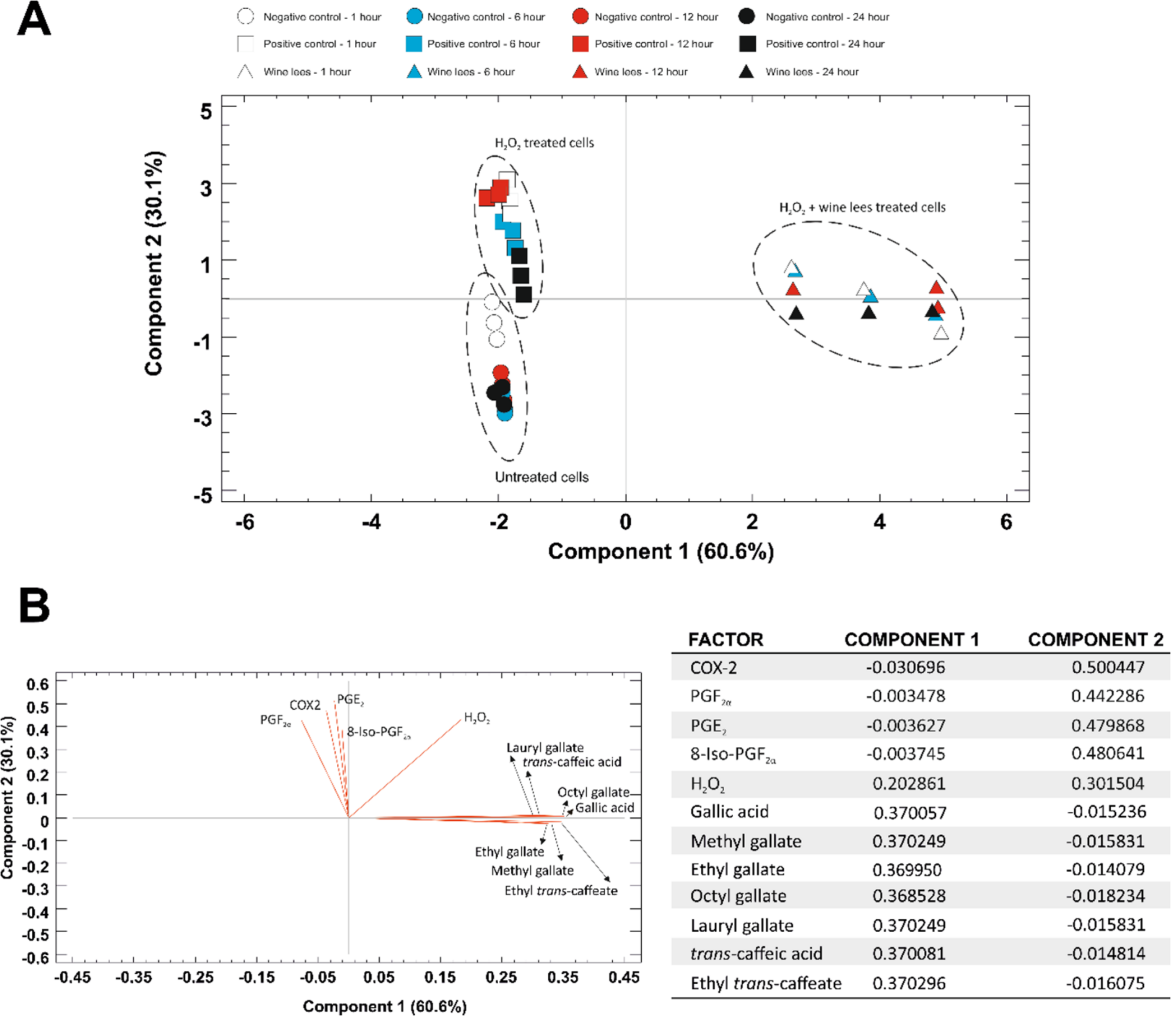
Principal component analysis (PCA) dot plot for the two principal components derived from the phenolic acids and phenolic esters of the bioaccessible fraction of wine lees, along with the inflammatory and oxidative stress markers (A). Principal component analysis scatter plot and loadings for the first two principal components (B).

PCA was applied to assess the contribution of phenolic alkyl esters to the prevention/resolution of OS and para-inflammation. This multivariate technique reduces the dimensionality of a data set, highlighting relationship patterns among various variables and associated specific compounds and bioactivities. Thus, in the present work, PCA was applied to alkyl gallates and caffeates in the bioaccessible fraction of wine lees and their ability to modulate the expression of COX-2, as well as the levels of 8-iso-PGF_2α_, PGF_2α_, and PGE_2_ (markers and mediators of OS and para-inflammation). As a result, acceptable scores were retrieved for the two first principal components (PC1 and PC2), which explained 90.7% of the variance (Figure 6). The clustering obtained showed three main groups that can be further understood alongside the PCA. In this plot, the cluster corresponding to untreated cells presented negative scores for both PC1 and PC2, indicating that it was characterized by both concentration of lipophenols (absent in these samples) and expression level of OS and para-inflammation markers and mediators, which remained in low levels as they did not receive the H_2_O_2_ pro-oxidant treatment. Alternatively, the cluster representing cells exposed to 50 μM H_2_O_2_, in the absence of bioaccessible lipophenols, correlated positively with the expression of markers and mediators of OS and inflammation, which increased significantly (positive scores for PC2) but displayed similar behavior to untreated cells concerning the level of galloyl and *trans*-caffeoyl alkyl esters (not present in these samples, thus displaying negative scores for PC1). The third cluster comprised samples pretreated with the bioaccessible fraction of gallic and *trans*-caffeic acid lipophenols. These samples exhibited positive scores for PC1, which were mainly associated with the concentration of such bioactive compounds. Concerning PC2, which represents the changes in the markers and mediators of the pathophysiological conditions monitored, lipophenols lowered the concentration of these indicators, reducing the positive scores displayed in H_2_O_2_-treated samples for PC2 to almost neutral values, according to the prevention of OS and anti-inflammatory effects developed by the galloyl and *trans*-caffeoyl alkyl esters (Figure 6B). In this regard, all phenolic lipophilized derivatives contributed similarly to the decrease of COX-2, 8-iso-PGF_2α_, PGF_2α_, and PGE_2_, with the highest values corresponding to octyl gallate and ethyl *trans*-caffeate (−0.018234 and −0.016075, respectively) (Figure 6B).

These findings, despite the valuable preventive activity of almost all gallic and *trans*-caffeic acid alkyl esters against OS and para-inflammation,^66^ are in good agreement with previous descriptions of the significant contribution of octyl gallate and ethyl *trans*-caffeate to the inhibition of inflammatory mediators by modulating the levels of COX-2 and derived prostanoids (PGF_2α_ and PGE_2_),^67,68^ extending this activity to OS marker 8-iso-PGF_2α_. Furthermore, the obtained results deliver further insight by providing evidence of the effectiveness of bioaccessible concentrations, which can have a closer connection to the physiological fraction responsible for the intended biological activities.

In conclusion, alkyl esters of gallic and *trans*-caffeoyl acids in wine lees could act as natural modulators of biological processes, preventing OS and inflammation associated with IBD. However, to date, there have been limited reports on these bioactivities concerning bioaccessible fractions (which involve severe modification of the phytochemical profile compared to the raw materials), making the discussion about the functionality of the bioaccessible profiles and concentrations relevant. In the frame of the overall existing evidence on the biological interest of alkyl esters of phenolic acids, the present work provides detailed insights into the bioaccessibility of alkyl gallates and *trans*-caffeates present in the referred enological residue. Thus, the present work provides comprehensive information about the relative contribution of the different gastrointestinal environments (physicochemical conditions and enzymatic activities) to the bioaccessibility of gallic and *trans*-caffeic acid alkyl esters. In this regard, the synthesis of new gallic and *trans*-caffeic acids lipophenols during gastrointestinal digestion supports the valorization of this matrix as a natural and sustainable source of these, recently characterized, bioactive compounds. In addition, beyond the lipophenolic content of wine lees, this matrix has special interest because it provides the molecular profile needed for synthesizing additional galloyl and *trans*-caffeoyl alkyl esters during gastrointestinal digestion, with the essential contribution of digestive enzymes. This evidence opens new valorization opportunities for this matrix, especially as a dietary source of bioactive lipophenols, due to the transformation suffered by its composition in the intestinal lumen with the aid of digestive enzymes.

The production of high amounts of bioactive phytochemicals in the intestinal lumen from wine lees prompted us to envisage the contribution of this byproduct to the prevention of inflammatory processes in this organic region. Thus, according to the results obtained, the galloyl and *trans*-caffeoyl alkyl esters of wine lees rendered bioaccessible concentrations effective against OS and para-inflammation, as evidenced by the modulation of corresponding markers and mediators associated with IBD. These outcomes demonstrated the potential of wine lees for developing nutraceuticals and functional ingredients and foods aimed at populations with identified risk factors for IBD. However, new challenges represented by the ability of wine lees’ lipophenols to cross the intestinal barrier and their subsequent metabolization need to be further elucidated to determine the potential of this matrix and its bioactive phytochemicals to achieve effective concentrations in distant organs and tissues, as well as to modulate additional mediators directly correlated with the activation of immune cells in autoreactive response within the context of inflammation.

## Data Availability

All data generated or analyzed during this study are included in this published article.

## Supporting Information Available

Multiple reaction monitoring (MRM) parameters, limits of detection (LODs), and limits of quantification (LOQs) of the separate phenolic acids and ester derivatives obtained by UHPLC–QqQ-MS/MS in the negative ionization mode (PDF)
