# Supplementary material for: Bioaccessible Phenolic Alkyl Esters of Wine Lees Decrease COX-2-Catalyzed Lipid Mediators of Oxidative Stress and Inflammation in a Time-Dependent Manner
Source: J Agric Food Chem. 2024 Aug 15;72(34):19016–27. doi: 10.1021/acs.jafc.4c05086 (PMC11363137; doi:10.1021/acs.jafc.4c05086)
Supplement: Supplementary file 1 — jf4c05086_si_001.pdf [file jf4c05086_si_001.pdf]

Supporting Information

Bioaccessible phenolic alkyl esters of wine lees decrease COX-2-catalyzed lipid mediators of oxidative stress and inflammation, in a time-dependent manner

Concepción Medrano-Padial; Irene Pérez-Novas; Raúl Domínguez-Perles*; Cristina García-Viguera; Sonia Medina

Laboratorio de Fitoquímica y Alimentos Saludables (LabFAS), CSIC, CEBAS, Campus Universitario de Espinardo 25, 30100, Espinardo, Murcia (Spain).

Supplementary Table 1. Multiple reaction monitoring parameters, limit of detection (LOD), and limit of quantification (LOQ) of the separate phenolic acids and ester derivatives obtained by UHPLC-QqQ-MS/MS in the negative ionization mode.

**Compound**	**Retention time (min)**	**Molecular weight**	**Precursor ion (*m/z* [M-H])**	**Product ion (*m/z* MS2[M-H])**	**Fragmentor (V)**	**Collision energy (eV)**	**Standard curve**	**Correlation Coefficient (R^2)**	**LOD (ng/mL)**	**LOQ (ng/mL)**
Gallic acid	0.52	170.12	169	151; **125**; 79	90	9	y = 194480x-4687	0.997	0.663	1.327
Methyl gallate (C1:0)[a]	0.67	184.15	183	169; **124**; 78	90	9	y = 443100x+4915	0.998	0.166	0.359
trans-Caffeic acid	0.74	180.16	179	**135**; 134; 107	90	9	y = 507260x+5043	0.999	0.702	1.405
Ethyl gallate (C2:0)	0.79	198.17	197	169; 151; **124**	90	9	y = 263196x+1449	0.999	0.178	0.386
Propyl gallate (C3:0)	1.06	212.20	211	169; **124**; 111	90	9	y = 1011649x+13448	0.999	0.191	0.414
Butyl gallate (C4:0)	1.39	226.23	225	169; **124**; 111	90	9	y = 792967x+12073	0.997	0.204	0.441
Ethyl *trans*-caffeate (C2:0)	1.44	208.21	207	179; 161; **135**	90	9	y = 408236x+3711	0.999	0.203	0.406
Octyl gallate (C8:0)	2.44	282.33	281	169; 140; **124**	90	9	y = 482642x+6466	0.997	0.254	0.551
Lauryl gallate (C12:0)	3.37	338.44	337	169; **124**; 111	90	9	y = 67460x+200	0.997	1.320	2.640

[a] The information in parentheses refers to the length of the alkyl chain linked to phenolic acid. Product ion (*m/z* [M-H]) in bold was used for quantification.
